# Correcting Artifacts in Single Molecule Localization Microscopy Analysis Arising from Pixel Quantum Efficiency Differences in sCMOS Cameras

**DOI:** 10.1038/s41598-019-53698-x

**Published:** 2019-12-02

**Authors:** Hazen P. Babcock, Fang Huang, Colenso M. Speer

**Affiliations:** 1 Center for Advanced Imaging, Harvard University, Cambridge, MA 02138 USA; 2 Weldon School of Biomedical Engineering, Purdue University, West Lafayette, IN 47907 USA; 3 Department of Biology, University of Maryland, College Park, MD 20742 USA

**Keywords:** Fluorescence imaging, Optical imaging, Super-resolution microscopy

## Abstract

Optimal analysis of single molecule localization microscopy (SMLM) data acquired with a scientific Complementary Metal-Oxide-Semiconductor (sCMOS) camera relies on statistical compensation for its pixel-dependent gain, offset and readout noise. In this work we show that it is also necessary to compensate for differences in the relative quantum efficiency (RQE) of each pixel. We found differences in RQE on the order of 4% in our tested sCMOS sensors. These differences were large enough to have a noticeable effect on analysis algorithm results, as seen both in simulations and biological imaging data. We discuss how the RQE differences manifest themselves in the analysis results and present the modifications to the Poisson maximum likelihood estimation (MLE) sCMOS analysis algorithm that are needed to correct for the RQE differences.

## Introduction

The high readout speed, high quantum efficiency, and relatively low cost of modern sCMOS cameras make their use attractive for SMLM. Recently they have started to displace Electron Multiplying Charge-Coupled Device (EMCCD) cameras in part because of their much greater bandwidth. A typical sCMOS camera can acquire 4 million pixels at a frame rate of 100 Hz, an order of magnitude faster than an EMCCD camera^1^. This speed, in combination with quantum efficiencies that are often greater than 80%, allows high throughput as well as live-cell SMLM with a large field of view^2–7^. However, this performance boost comes with the disadvantage of much greater pixel-to-pixel variability in comparison with traditional CCD cameras mandating statistical treatment of these pixel-dependent noise in single molecule imaging experiments^6^ as well as general microscopy^8^. The first demonstration of such treatment in single molecule localization analysis focused on handling differences in gain, offset and readout noise^6^.

As the dominant pixel-to-pixel variations are found in the readout noise, offset, and gain it is reasonable to focus on these properties first. However, we found that it is also necessary to compensate for differences in the RQE of individual pixels for the sCMOS sensors that we tested in this work. For example, in the two cameras that were tested the pixel-dependent RQE varied by ~4% over a relatively small region of 10 × 10 pixels. These rapid variations are large enough to lead to measurable systematic errors in single molecule identification and fitting. At the single molecule identification step a single-valued threshold on localization height or integrated pixel intensity (sum) is often employed as part of the process of deciding whether or not the identified pixels correspond to a single molecule emission or noise. Such a threshold biases the localization identification against regions of the camera with lower RQE, an effect that is particularly noticeable for data with a lower signal-to-noise ratio. In addition, at the localization fitting step, the variations are large enough to lead to systematic errors on the order of several nanometers (shown below) in the localization of single molecules, creating a significant source of error for ultra-high precision SMLM based techniques^9,10^.

This work discusses the corrections necessary to the Poisson MLE sCMOS algorithm described in Huang *et al*.^6^ to handle pixel-dependent RQE differences. Other labs have also presented work that discusses correcting for the pixel-dependent differences in offset, gain, variance and quantum efficiency (QE) for sCMOS cameras for optimal weighted least squares fitting^11,12^. In these works the authors use the average camera gain as the gain value for each pixel. Then any pixel-dependent differences from the average gain and the average QE are combined into a single pixel-dependent term, a flat field correction factor. This approach works well for least squares fitting, which is the maximum likelihood estimator for Gaussian distributed data, because for a Gaussian distribution the mean and variance are independent. It can however be problematic for MLE fitting of Poisson distributed data, where the variance is expected to be equal to the mean. If the camera has substantial pixel level differences in QE the result from using the average gain and a flat field correction term to convert camera values (ADU) to photo-electrons (e-) will no longer be a Poisson distribution with mean equal to the variance. Because this can degrade fitting performance when the fitter assumes Poisson statistics, we instead use a pixel-dependent gain value that preserves the expected relation between mean and variance and a pixel-dependent RQE term to compensate for any differences in pixel QE. We focus on this problem as it has been shown that weighted least squares fitting is not as accurate as MLE fitting on Poisson distributed data, especially at the lowest signal levels^13,14^.

## Results

sCMOS camera calibration data were acquired following the procedure described previously^6^. Movies that were 20k frames in length were acquired at 4 different illumination intensities and in the dark. These movies were used to characterize the gain (*g*_*i*_), offset (*o*_*i*_), read noise variance (*var*_*i*_), and RQE (*rqe*_*i*_) of each pixel on the camera. First the offset and the read noise variance were determined for each pixel by measuring the mean and the variance versus time of camera frames obtained in the dark. Then the pixel temporal mean and variance was measured at each illumination level. The measured means and variances at each level were corrected by subtracting the means and variances of the dark condition, subsequently referred to as the corrected means and variances. The gain for each pixel was determined from the slope of a line fit to the corrected mean and variance values as well as a point at the origin. We did not observe non-linearities in the mean versus variance curve that were reported in^12^ over the range of illumination intensities used in this work. In the fitting, equal weights were given to every point. Finally, the temporal means in the dataset at the highest illumination level were converted to e- using the characterized gains and offsets. A smoothed version of the converted temporal means was created by convolution with a uniform filter of size 10 × 10 pixels (using scipy.ndimage.uniform_filter()^15^). The RQE for each pixel was calculated by dividing the means by the smoothed version of the means. The local uniform filter was used to reduce the effect of any long-range non-uniformities in illumination across the field of view (See Methods Section: Camera Calibration).

**Figure 1:**
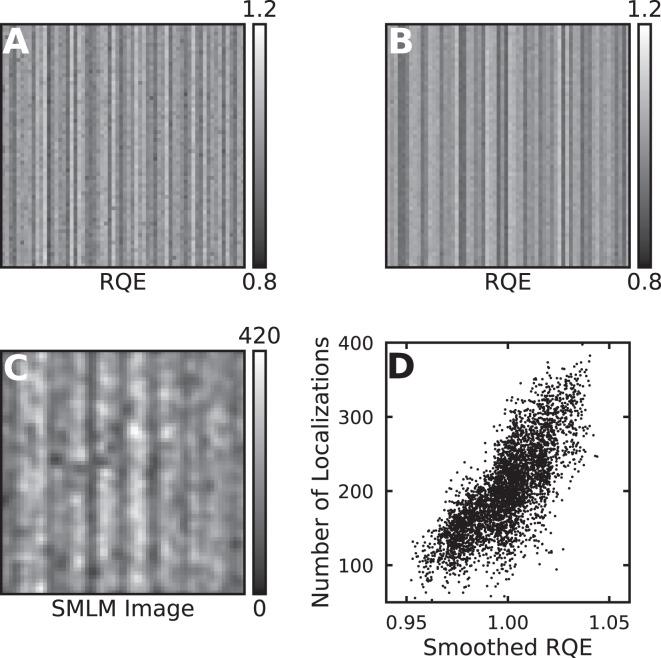
sCMOS camera RQE maps and analysis artifacts in simulated data. (**A**) The measured gain for each pixel in a 64 by 64 pixel region of a sCMOS camera. (**B**) Same as (**A**) for a different sCMOS camera. (**C**) SMLM image resulting from the analysis of simulated SMLM movie. This image was created by rendering the localizations as Gaussians with unit height and a sigma equal to 1 pixel. A pixel itensity value of 420 corresponds to approximately 420 localizations found in or very near the pixel. The movie was analyzed assuming a constant RQE value of 1.0. The measured sCMOS calibration data shown in (**A**) was used to create the simulated SMLM movie as described in the main text. The image is 6.4 × 6.4 μm with a 100 nm pixel size (**D**) A scatter plot of the number of localizations identified in each pixel versus the pixels smoothed RQE value.

An image of the camera RQE for each pixel in a 64 × 64 pixel sub-region of two different camera chips is shown in Fig. 1A,B. The standard deviation of the pixel RQEs is ~4%. This is substantially larger than the uncertainty in the measurement, which is ~1% (Supplementary Fig. S1). We also observed that the pixel gain values have a standard deviation of ~4%, and that the gain values are anti-correlated with the RQE values, that is pixels with a higher RQE value have a lower gain value. The anti-correlation is obvious in 2D histograms of the gain versus RQE (Supplementary Fig. S2). While balancing these two terms makes for a more visually uniform image we reasoned that it might be problematic for sCMOS localization algorithms that assume that every pixel has the same RQE.

To explore this issue a measured sCMOS calibration was used to simulate SMLM movies of uniform randomly distributed emitters at an average density of 0.15 emitters per μm^2^. The emitters turned on and off stochastically with an off-rate of 2 frames. When on, emitters generated photons at a constant rate of 250 photons per frame. In addition we introduced a uniform background of 20 photons per pixel. The photon counts in each pixel were multiplied by the measured RQE values and then used as the the expectations for the creation of Poisson random distributed e- values. These e- were multiplied by the camera gain values, and then Gaussian distributed random variables with standard deviations corresponding to the camera pixel-dependent read noise were added. Finally the camera offset values were added to create the simulated sCMOS measured camera image (example images shown in Supplementary Fig. 3A,C).

When these data were analyzed assuming a constant RQE of 1.0 we observed a striking stripe pattern artifact in the SMLM image (Fig. 1C). The emitters were uniformly distributed so the correct analysis result is an essentially featureless SMLM image. Instead the SMLM image was highly correlated with the camera RQE values (camera RQE values for comparison are shown in Fig. 1A). This correlation is even more obvious in Fig. 1D, where the number of localizations that were found in any given pixel is plotted against the smoothed RQE of the pixel. The sCMOS analysis algorithm that we used employs the SNSMIL^16^ approach adapted for sCMOS localization identification. In our algorithm the noise in the image is reduced by smoothing with a Gaussian kernel with a sigma equal to the expected PSF sigma. Because of this smoothing, localization identification is only sensitive to RQE differences after smoothing with the kernel, which is what we refer to as smoothed RQE in the text and figure captions. We observed that 3–4x more localizations were found centered on pixels with a high RQE (after smoothing). This artifact is most striking when the average localization intensity is close to the threshold value for deciding whether a localization is real or a noise artifact, as was the case here. For data where the majority of the localizations are well above this threshold the RQE effects on localization identification are less obvious, but will still be present due to the stochastic nature of fluorescent dye emission in SMLM (data not shown).

1 $${x}_{pre,i}=\frac{({x}_{raw,i}-{o}_{i})}{{g}_{i}}$$

2 $${\chi }_{MLE}^{2}=2\mathop{\sum }\limits_{i=1}^{N}\,({f}_{i}-{x}_{i})-2\mathop{\sum }\limits_{i=1}^{N}\,{x}_{i}\,{ln}(\frac{{f}_{i}}{{x}_{i}})$$

3 $$\begin{array}{rcl}{f}_{i} & = & f(\theta )+va{r}_{i}\\ {x}_{i} & = & {x}_{pre,i}+va{r}_{i}\end{array}$$

4 $$f(\theta )={\theta }_{1}\ast \exp (\frac{-({(x-{\theta }_{2})}^{2}+{(y-{\theta }_{3})}^{2})}{(2\ast {\sigma }^{2})})+{\theta }_{4}$$

**Figure 2:**
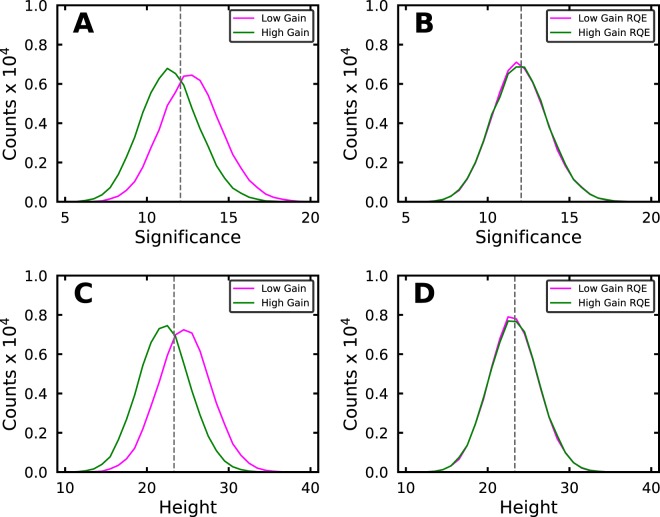
Compensation for RQE effects when identifying localizations in simulated data. (**A**) Histograms of localization (SNSMIL) significance without RQE correction. The “low gain” category (magenta line) includes localizations that were centered on pixels that had gain values in the bottom 20% of the smoothed gain value distribution. The “high gain” category (green line) is the same except it is the pixels that had gain values in the top 20%. The gray dashed line is the mean localization significance value. (**B**) Same as (**A**) after RQE compensation. (**C**) Histograms of localization heights without RQE correction. Categories are as in (**A**). (**D**) Same as (**C**) after RQE compensation.

The analysis algorithm pre-processes the camera image *x*_*raw*,*i*_ using (1) prior to the localization identification step. When both the gain and RQE vary from pixel-to-pixel in the anti-correlated manner observed here the effect of (1) is to suppress *x*_*i*_ in regions of high gain (and low RQE). This suppression is the likely source of the artifacts in the localization identification shown in Fig. 1. To test if this was indeed the case we created simulated sCMOS camera data as described above. In this simulation however the emitters were on in every frame with a constant intensity of 250 photons per frame, instead of stochastically blinking as in the previous simulation. In addition the emitters were placed on grid with a 20 pixel separation between neighboring emitters instead of being uniformly distributed across the image. In total we simulated 300k emitters in a 1k frame movie with 300 emitters per frame. We fit each emitter (a localization) with a 2D Gaussian with a fixed sigma of 1.5 pixels (100 nm pixel size) ((4)) using a variation of the Levenberg-Marquadt algorithm^17,18^ to minimize the Poisson MLE *χ*^2^ shown in (2). The fitting function (*f*_*i*_) and data (*x*_*i*_) terms in Eq. (2) include sCMOS pixel read noise variance correction terms (see Eq. (3)) as described in Huang *et al*.^6^. Once we determined the best fit *θ* for each localization we calculated its significance. Significance is the SNSMIL metric that describes the probability that a localization is real in units of sigmas from the mean. The noise value for a localization is estimated as the square root of the number of e- in the background, which is based on the assumption of Poisson statistics where the variance equals the mean. Then the localization’s significance is the number of e- in the background subtracted localization divided by it’s noise value. In theory the probability that a localization with a significance of 6 sigma is just a noise artifact is extremely small (~2.0 * 10^−9^ assuming Gaussian statistics). Then we grouped the localizations into categories based on the smoothed gain value of the pixel they were centered on. Smoothed gain is similar to smoothed RQE described above, the measured camera gain values are convolved with the same Gaussian kernel that was used for localization identification to calculate the smoothed gain values. In Fig. 2A histograms of the significance are plotted for the localizations that were centered on pixels in the lowest (“low gain”) or highest (“high gain”) 20% of all of the pixel smoothed gain values. A detection threshold with a value equal to that of the gray dashed line in Fig. 2A is clearly biased against “high gain” localizations. This threshold would discard 60% of the localizations in the “high gain” category, but only 40% of the localizations in the “low gain” category. The best fit localization height (*θ*_1_) is another metric that commonly used for localization thresholding. However a threshold based on this metric would be biased in a manner that is identical to that based on significance as shown in Fig. 2C.

5 $${x}_{pre,i}=({x}_{raw,i}-{o}_{i})/({g}_{i}\ast rq{e}_{i})$$

We found that dividing the camera image *x*_*raw*,*i*_ by *rqe*_*i*_ as shown in (5) greatly reduced the dependence of localization significance on pixel RQE and gain differences. Using (5) to pre-process the camera images we re-ran the analysis on the same simulated data that we used for Fig. 2A,C. After this change the histograms of localization significance and height for the “low gain” and “high gain” categories are now virtually indistinguishable (Fig. 2B,D). A detection threshold with a value equal to that of the gray dashed line in Fig. 2B,D is no longer biased against localizations in the “high gain” versus the “low gain” categories.

6 $$\begin{array}{rcl}{f}_{i} & = & f(\theta )rq{e}_{i}+{\mathrm{var}}_{i}\\ {x}_{i} & = & ({x}_{raw,i}-{o}_{i})/{g}_{i}+{\mathrm{var}}_{i}\end{array}$$

7 $$\begin{array}{rcl}{f}_{i} & = & f(\theta )\\ {x}_{i} & = & \frac{({x}_{raw,i}-{o}_{i})}{fla{t}_{i}\ast  < {g}_{i} > }\end{array}$$

8 $${\chi }_{WLS}^{2}=\mathop{\sum }\limits_{i=1}^{N}\,\frac{{({f}_{i}-{x}_{i})}^{2}}{{x}_{i}+{\mathrm{var}}_{i}}$$

9 $$fla{t}_{i}=\frac{({g}_{i}\ast rq{e}_{i})}{ < {g}_{i} > }$$

Next, we characterized the effects of the pixel-dependent RQE on localization fitting and explored alternative possible corrections to compensate for RQE-dependent effects on fitting accuracy. To do this we performed additional simulations using the previously described sCMOS calibration. These simulations had a constant background of 20e- per pixel and a range of different emitter intensities designed to span values that are encountered in SMLM experiments. The emitters were separated by 20 pixels from each other on a 2D grid pattern so that neighboring fits would not affect each other. Three different fitting methods were performed on the simulated data. The first, “No Correction”, is a Poisson MLE fit that minimizes *χ*^2^ in Eq. (2), and that uses Eq. (1) for image pre-processing and Eq. (3) for pixel read noise variance correction. It is referred to as “No Correction” because it assumes that the RQE values are all equal to 1.0. The second approach, “WLS Flat Field”, performs a weighted least squares fit on flat-field corrected images following^11,12^. In this analysis the offset was first subtracted from the raw camera data, then the result was divided by the product of the average camera gain value and a flat-field correction term (shown in Eq. 7). The flat-field correction term was calculated using Eq. (9). Finally the processed image was fit by minimizing *χ*^2^ in Eq. (8). The final approach “RQE Correction” is very similar to “No Correction”, but now includes both read noise variance and RQE correction as shown in Eq. (6). This approach is more complicated than only including the RQE term in the image processing step (Eq. (5)) as we recommended for localization identification, but it is more accurate because it does not distort the Poisson statistics of the image.

**Figure 3:**
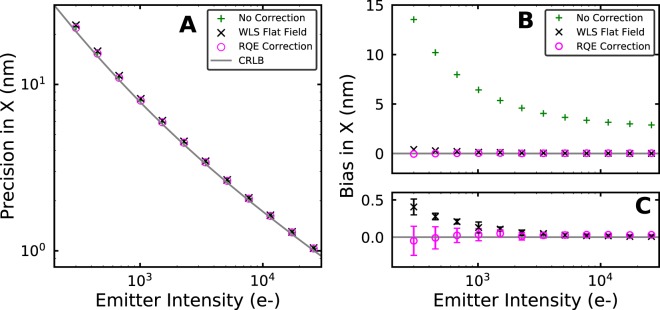
A graph of the effects of differences in RQE on fitting precision and fitting bias as measured by fitting simulated data. In the “Uncorrected” approach the RQE values were all set to 1.0 in the fitting. In the “WLS Flat Field” all the pixels had the same gain value, the average of the pixel gain values, the images were flat-field corrected and fitting was done using weighted least squares fitting. In the “RQE Correction” approach the fit included the known RQE and gain values for each pixel. CRLB is the Cramer-Rao lower bound calculated as described in Methods. (**A**) Fitting precision versus emitter intensity. (**B**) Magnitude of the bias in fitting versus emitter intensity. (**C**) Same as (**B**) but only showing the “WLS Flat Field” and “RQE Correction” results (see legend in **B**). Error bars are standard deviations estimated from 10 independent simulations for each data point.

The performance of the different fitting approaches as compared to the theoretical limit is shown in Fig. 3. We calculated the theoretical limit of fitting precision using the Cramer-Rao lower bound (CRLB) formalism as described in^13,19^ modified to include the effects of pixel-dependent differences in read noise variance (see Methods). In order to calculate fitting precision we used 1000-frame long simulated movies and calculated the standard deviation from the measured mean position for each emitter. The precision values from 126 different emitters were averaged and are shown in Fig. 3A. All of the different fitting approaches had very similar precision values that were also all quite close to the CRLB. We also examined the bias in each fitting approach by measuring the root mean square of the difference between the measured mean position of each emitter and it’s ground truth position, corrected for measurement precision. We found that the “RQE Correction” fitting approach is an unbiased estimator of the ground truth emitter position, with bias values that are within the estimated error of zero (Fig. 3B,C). In this figure the error bars are the standard deviation estimated from the results from 10 independent replicates of the simulations. The “WLS Flat Field” fitting approach appears to have a sub-nanometer bias at the lowest emitter intensities studied, but it is not clear if the bias is large enough to be statistically significant (>6 sigma). The “No Correction” fitting model however, which ignores RQE differences, has a large bias ranging from 2 nm to 13 nm depending on emitter intensity. This is a substantial amount of bias as it ranges from 50% of the precision value at the lowest intensity to 250% of the precision value at the highest intensity. The bias in fitting is primarily in the X coordinate due to the vertical orientation of the striped RQE pattern (Fig. 1A,B)^20^.

**Figure 4:**
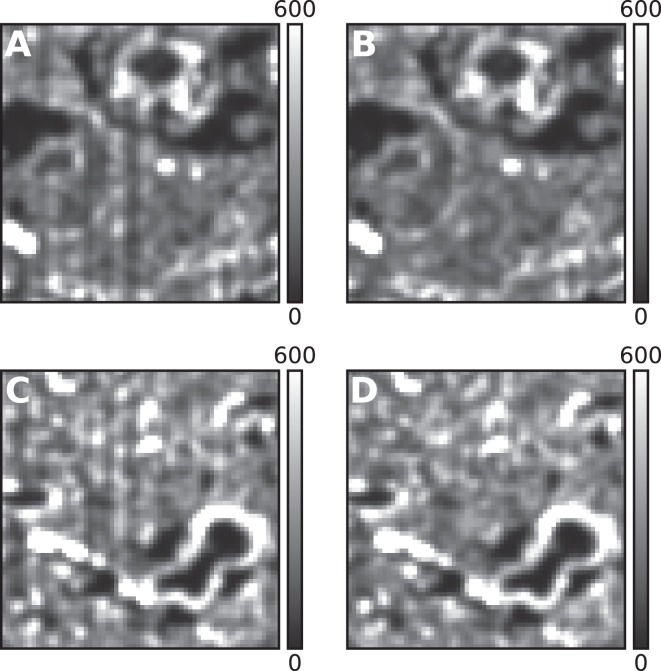
Images of mouse brain tissue stained with wheat germ agglutinin conjugated to ATTO-488, embedded in epoxy resin and sectioned at 70 nm thickness. (**A**) SMLM image of part of a tissue section analyzed without RQE correction showing evidence of a vertical striping artifact. (**B**) Same as (**A**) but analyzed with RQE corrected localization identification and fitting. (**C**) SMLM image of the same camera ROI shown in (**A**) but of a different physical tissue section. (**D**) Same as (**C**) but analyzed with RQE-corrected localization identification and fitting. All images were created by rendering the localizations as Gaussians with unit height and a sigma equal to 1 pixel. A pixel intensity value of 600 corresponds to approximately 600 localizations found in or very near the pixel. Images are 9.8 × 9.8 μm and the pixel size is 153 nm.

Finally we explored the effects of RQE differences on the analysis of experimental data. To do this we acquired 20k-frame long SMLM movies of glycoproteins in brain tissue stained using wheat germ agglutinin (WGA) conjugated to ATTO-488. Following staining, the tissue was dehydrated, embedded in an epoxy resin, and cut into 70 nm serial physical sections using an ultra-microtome. The sample was illuminated with a 488 nm laser at a power density of 4.4 kW/cm^2^ and images were acquired at a frame rate of 60 Hz with the sCMOS camera whose RQE map is shown in Fig. (1B). Several different SMLM movies of WGA staining in separate physical tissue sections were acquired and analyzed with and without RQE correction. SMLM images of ROIs of two of these movies are shown in Fig. (4). Both of these ROIs are from exactly the same region of the camera which is also the same ROI as shown in Fig. (1B). Comparing Fig. (4A,C), which show the final SMLM images of two different tissue sections without RQE correction, both ROIs have the same striped artifact indicating that the artifact is camera ROI-dependent and is not based on the biological features of the sample. This artifact is most obvious with dimmer dyes, such as the ATTO-488 dye used here, and is less obvious with emitters that emit higher numbers of photons per frame (data not shown). On average the intensity of single ATTO-488 emitters is only ~1.4x larger than the threshold value used for separating real localizations from noise. The striped artifact was no longer observed when RQE correction as described above was included in the analysis (Fig. (4B,D)). We also calculated the Pearson correlation coefficient between the number of localizations centered over any given pixel and the smoothed RQE value of the pixel. When the analysis was not corrected for pixel-to-pixel RQE differences, the probability the correlation was random was less than 10^-13^, but when RQE correction was included the probability that the correlation was random increased to ~0.2. This indicates that the corrections we propose are successfully compensating for pixel-dependent RQE differences.

## Discussion

In this work, we present the modifications to the Poisson MLE sCMOS algorithm necessary to correct for the significant pixel-to-pixel RQE differences encountered in some sCMOS cameras. The first modification is to include the pixel RQE term in the image pre-processing step using Eq. (5) in order to remove the RQE-dependent bias in localization identification. The second modification is to include the pixel RQE term in the fit function as shown in Eq. (6). This modification restores CRLB fitting performance. These modifications have been included in sCMOS analysis package of the open source storm-analysis project^21^ that is available on Github.

## Methods

### Microscope setup

The setup is based on an inverted microscope (TiU, Nikon) mounted on an optical table (RS2000, Newport). This microscope has a brightfield lamp and condenser (Ti-C-LWD 0.52, Nikon) which was used for camera calibration. The sCMOS camera (ORCA-Flash4.0 v2, Hamamatsu Photonics) was mounted directly onto the left port of the microscope with no additional magnification.

### Camera calibration

A 10x 0.3NA air objective (CFI Plan Apo Lambda 10x 0.45NA, Nikon) was used without any sample mounted on the microscope for calibration. This geometry provided illumination that was uniform at the 10% level across the FOV (512 × 512 pixels). The illumination was greatest near the center and decreased smoothly towards the edges of the FOV. When measured across 10 × 10 pixel sub-regions for the FOV, as was done for the RQE measurement, the variation in illumination intensity was reduced to approximately 1.7%.

The stability of the intensity of the brightfield lamp was measured by taking the average intensity of each frame of the calibration movie. Contributions to the pixel variance due to fluctuations in the intensity of the brightfield lamp were ~5% at the highest intensity used in calibration. Though small, these fluctuations were corrected for by subtracting the variance of the average intensity from the variance for each pixel.

### sCMOS camera terminology

Offset *o*_*i*_ is the average intensity of each pixel *i* of the camera when no photons are incident on the camera.

10 $${o}_{i}=\frac{1}{M}\mathop{\sum }\limits_{m=1}^{M}\,{s}_{i}^{m}$$

where *s*_*i*_^*m*^ is the ADU count at frame *m* for pixel *i* and M is the total number of dark frames acquired.

Variance *var*_*i*_ is the variance of each pixel *i* of the camera when no photons are incident on the camera.

11 $${\mathrm{var}}_{i}=\frac{1}{M}\mathop{\sum }\limits_{m=1}^{M}\,{({s}_{i}^{m})}^{2}-{o}_{i}^{2}$$

Gain *g*_*i*_ is calculated by least squares minimization of the following equation.

12 $${\hat{g}}_{i}={argmin}\mathop{\sum }\limits_{k=0}^{K}\,{(({V}_{i}^{k}-{\mathrm{var}}_{i})-{g}_{i}({D}_{i}^{k}-{o}_{i})+{b}_{i})}^{2}$$

where *V*_*i*_^*k*^ is the pixel temporal variance and *D*_*i*_^*k*^ is the pixel temporal mean of a movie taken at illumination level *k*. A *k* = 0 point is included in the fit where *V*_*i*_^*k*^ and *D*_*i*_^*k*^ are both equal to 0. The *b*_*i*_ term is also fit at the same time and allows for a nonzero offset.

RQE *rqe*_*i*_ is the corrected image at the highest illumination level divided by a smoothed version of the same image.

13 $$\begin{array}{rcl}i{m}_{i} & = & ({s}_{i}^{k}-{o}_{i})/{g}_{i}\\ s{m}_{i} & = & i{m}_{i} \circledast U(10x10)\\ rq{e}_{i} & = & \frac{i{m}_{i}}{s{m}_{i}}\end{array}$$

where *U*(10*x*10) is the 10 × 10 pixel uniform distribution normalized to sum to unity and $\circledast$ is the symbol for convolution.

### Cramer-Rao lower bound calculation

The Fisher information matrix is given by 14 where *θ* are the fitting parameters.

14 $$\begin{array}{llll}{F}_{n,m} & = & E[\frac{\partial \,\mathrm{ln}(L(\theta |D))}{\partial {\theta }_{n}}\cdot \frac{\partial \,\mathrm{ln}(L(\theta |D)}{\partial {\theta }_{m}}] & \\ \theta  & = & \theta (bg,h,{x}_{o},{y}_{o}) & 2{\rm{D}}\,{\rm{fixed}}\,{\rm{width}}\,{\rm{Gaussian}}\\ \theta  & = & \theta (bg,h,{x}_{o},{y}_{o},\sigma ) & 2{\rm{D}}\,{\rm{variable}}\,{\rm{width}}\,{\rm{Gaussian}}\end{array}$$

For a Poisson process with independent pixels (*i*) we can use the Stirling approximation to simplify 14 to 15. In this equation *μ*_*i*_(*θ*) is the fitting model.

15 $${F}_{m,n}=\mathop{\sum }\limits_{i=1}^{N}\,\frac{1}{{\mu }_{i}(\theta )}\frac{\partial {\mu }_{i}(\theta )}{\partial {\theta }_{n}}\frac{\partial {\mu }_{i}(\theta )}{\partial {\theta }_{m}}$$

With the RQE and sCMOS noise modifications *μ*_*i*_(*θ*) is given by 16.

16 $${\mu }_{i}(\theta )=rq{e}_{i}\ast f(\theta )+{\mathrm{var}}_{i}$$

Finally substituting this into 15 gives 17.

17 $${F}_{m,n}=\mathop{\sum }\limits_{i\mathrm{=1}}^{N}\,\frac{rq{e}_{i}^{2}}{rq{e}_{i}\ast f(\theta )+va{r}_{i}}\frac{\partial f(\theta )}{\partial {\theta }_{n}}\frac{\partial f(\theta )}{\partial {\theta }_{m}}$$

The Cramer-Rao bounds are then calculated from the inverted *F*_*m*,*n*_ matrix.

18 $$\mathrm{var}iance({\theta }_{m})\ge {F}_{m,m}^{-1}$$

This calculation is slightly more complicated than the CRLB calculation in^13,19^ because the bounds now depend on the localizations position in the image. The CRLB values used in the figures are the average CRLB of all the simulated localization positions.

### Simulations and analysis

All simulations and analysis were done using the open source storm-analysis project^21^. This project provides a mixture of Python and C language code that implements many of the common tasks in SMLM movie analysis.

### Tissue preparation and labeling for STORM

Animal work was performed in accordance with Institutional Animal Care and Use Committee (IACUC)-approved protocols at the University of Maryland, College Park. Tissue from a 6-week-old, WT C57BL/6J mouse (The Jackson Laboratory, stock #000664) was used to generate the data presented in Fig. (4). Samples were prepared in a manner similar to that previously described in Sigal *et al*.^22^. Briefly, the animal was transcardially perfused with 4% PFA (Electron Microscopy Sciences) in sterile 0.9% NaCl, the overlying cortex was removed from the brain, and the thalamus was removed and post-fixed by immersion in 4% PFA in sterile 0.9% NaCl for 1 hour at room temperature (RT). 100 μm vibratome sections were cut in the coronal plane of section on a VT1000S vibratome (Leica Microsystems Inc.). The dorsal lateral geniculate nuclei (dLGN) were identified by visual landmarks and circular punches ~500 μm in diameter containing the dorsal pole of the dLGN were removed bilaterally from brain sections using a blunt end needle. Tissue punches were blocked for ~4 hours at RT in a 1x phosphate buffered saline (PBS) solution containing 10% normal donkey serum (Jackson ImmunoResearch Laboratories Inc., product # 017-000-121), 0.3% Triton X-100 (Sigma-Aldrich Inc.), and 0.02% sodium azide (Sigma-Aldrich Inc.). Following blocking, tissue punches were incubated overnight in a 10:1 dilution of ~0.3 mg/ml wheat germ agglutinin (Vector Laboratories Inc., product # L-1020) conjugated to ATTO488-NHS ester (ATTO-TEC GmbH; product # AD 488-31) in 1x PBS solution. As previously described^22^, punches were washed, post-fixed, dehydrated in a graded series of ethanol, and infiltrated with UltraBed epoxy resin (Electron Microscopy Sciences, product # 14310) prior to polymerization (~16 hours at 70C). Polymerized tissue blocks were cut into ultrathin (70 nm) serial-section arrays using an Ultracut EM UC7 Ultramicrotome (Leica Microsystems Inc.) and sections were collected on #1.5 coverslips coated with 0.5% gelatin/0.05% chromium potassium sulfate (Sigma-Aldrich Inc.) and heated for ~25 minutes at 60C. Prior to imaging, coverslips containing tissue sections were chemically etched in 10% sodium ethoxide solution for 5 minutes. Coverslips with etched sections were secured to a glass slide using double-sided tape to create a flow chamber, which was filled with imaging buffer (10% glucose/17.5 mM glucose oxidase/708 nM catalase/10 mM MEA/10 mM NaCl/200 mM Tris) and sealed with fast-drying epoxy resin.

### STORM imaging

STORM images were collected on an Eclipse Ti-U inverted microscope using a Plan Apochromat 1.4 NA 60x oil-immersion objective (CFI Plan Apo Lambda 60x 1.4NA, Nikon Instruments Inc.). The microscope was fitted with a custom pentaband dichroic mirror and notch filter (Chroma Technology Corp.). For excitation, a high-power continuous-wave 488 nm laser (Genesis CX488, Coherent Inc.) was delivered to the sample via beam steering optics designed for oblique incident angle illumination. In the emission path, a custom c-mount containing a motorized filter wheel (HF110, Prior Scientific) equipped with emission filters (Semrock Inc.) was inserted immediately prior to a 0.7x relay lens, which was used to create a pixel size of ~153 nm at the imaging plane on a sCMOS camera (ORCA-Flash4.0 V3, Hamamatsu Photonics). The image pixel size was determined by measuring the distance between the fiducial lines in brightfield images of a stage micrometer slide (R1L3S2P, Thorlabs Inc.) using FIJI^23^. The STORM imaging field size was 640 × 640 pixels (~98 × 98 μm). Axial focus was maintained by a custom focus lock system with a 50/50 beamsplitter (Thorlabs Inc.) and steering optics to split a 980 nm fiber laser (LP980-SF15, Thorlabs Inc.) into two separate, laterally offset spots that are directed to the sample and reflected off the coverslip surface. IR spot reflections were monitored by relay optics imaging the beams onto a USB camera (DCC1645C, Thorlabs Inc.). Any deviation of the spots relative to their initial set position initiates a digital feedback signal to a nanopositioning piezo Z-stage (NZ400CE, Prior Scientific), which moves the sample in the axial plane to restore the set beam positions. STORM images of WGA-ATTO-488 were collected at 60 Hz for 20,000 frames each.

### Preprint

A preprint version of this paper was released on bioRxiv^24^.

## Supplementary information

Supplementary figures

## Data Availability

Camera calibration files and Jupyter^25^ notebooks that perform the simulations and create the figures are available from Zenodo (10.5281/zenodo.3546209). Data in this paper is available by request.
