# Supplementary material for: Correcting Artifacts in Single Molecule Localization Microscopy Analysis Arising from Pixel Quantum Efficiency Differences in sCMOS Cameras
Source: Sci Rep. 2019 Dec 2;9:18058. doi: 10.1038/s41598-019-53698-x (PMC6889274; doi:10.1038/s41598-019-53698-x)
Supplement: Supplementary file 1 — Supplementary figures [file 41598_2019_53698_MOESM1_ESM.pdf]

Correcting Artifacts in Single Molecule Localization Microscopy Analysis Arising from Pixel Quantum Efficiency Differences in sCMOS Cameras

Hazen P. Babcock[1,*], **Fang Huang**[2], **and Colenso M. Speer**[3]

[1]Center for Advanced Imaging, Harvard University, Cambridge, MA, 02138, USA
[2]Weldon School of Biomedical Engineering, Purdue University, West Lafayette, IN, 47907, USA
[3]Department of Biology, University of Maryland, College Park, MD 20742, USA
[*]hbabcock@fas.harvard.edu

Supplementary Figure S1 : sCMOS Calibration Repeatability

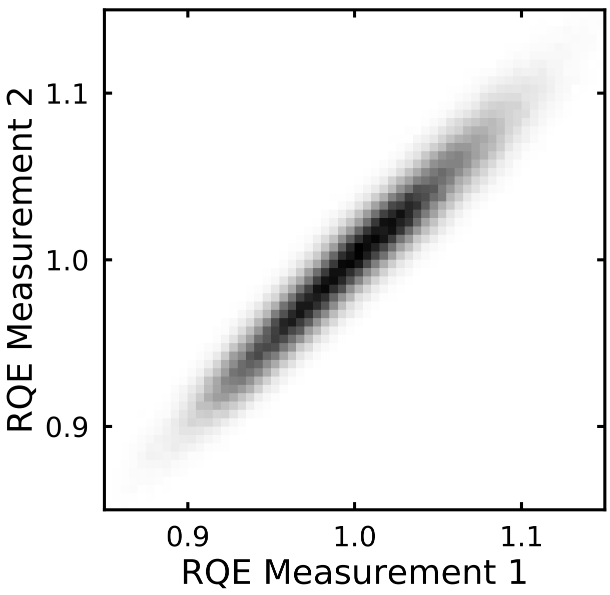

Figure S1. A 2D histogram of the RQE values measured in two different experiments to test the repeatability of the camera calibration measurements.

Supplementary Figure S2 : The Correlation between Gain and Relative QE

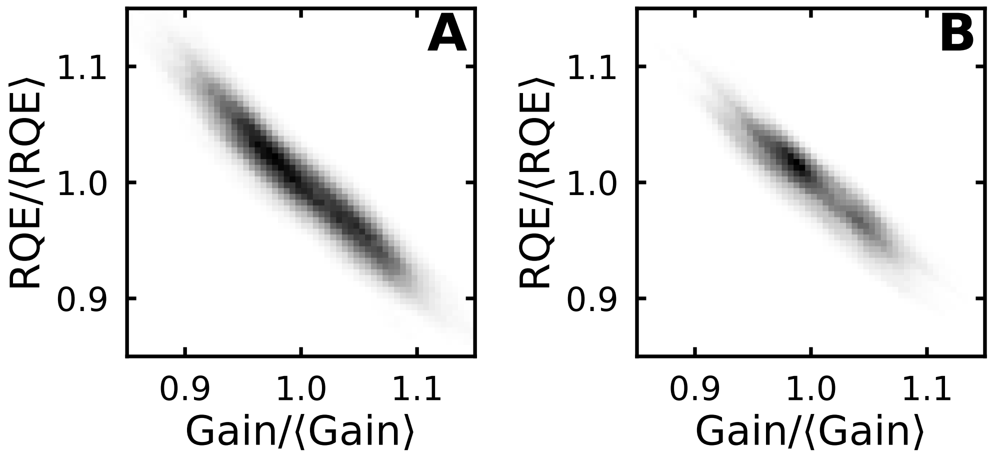

Figure S2. A 2D histogram of the normalized gain versus the normalized relative QE for each pixel. The strongly anticorrelated nature of these two values indicates that the gain is adjusted to compensate for differences in the relative quantum efficiency (RQE) of each pixel. (a) Camera 1. (b) Camera 2.

Supplementary Figure S3 : sCMOS Simulations Example Images

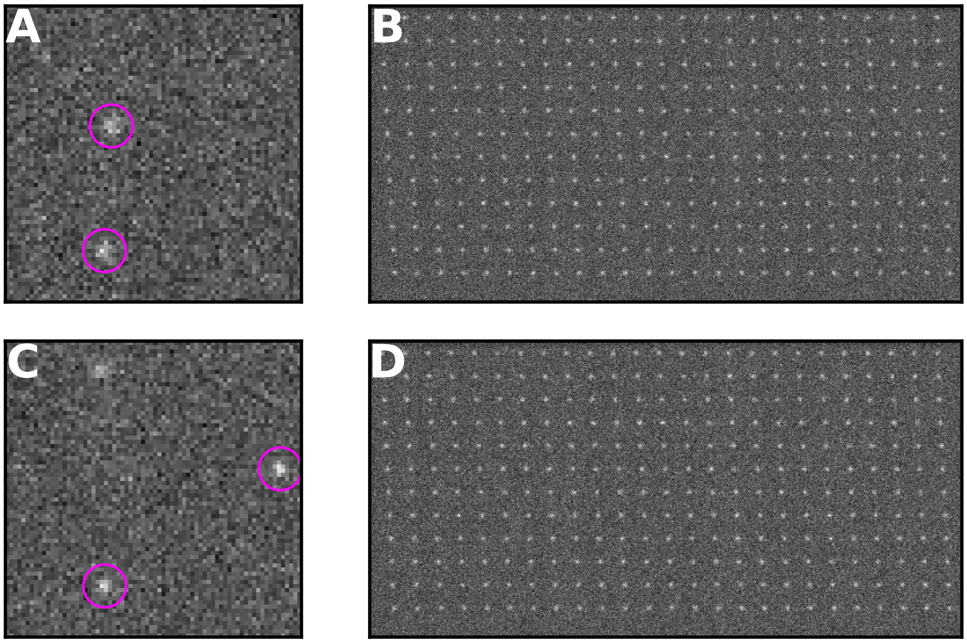

Figure S3. Example simulated images. (a, c) Example simulated SMLM images for testing the effect of RQE on localization identification. In these images the emitters were uniform randomly distributed and switched on and off stochastically. Magenta circles indicate localizations identified by the analysis. (b, d) Example simulated SMLM images that were fit to measure localization significance and height. The emitters are distributed on a grid and are on in every frame. Every emitter was identified and fit in this simulation so the magenta circles are not shown.
